# Radiation pneumonitis in patients with non-small-cell lung cancer receiving chemoradiotherapy and an immune checkpoint inhibitor: a retrospective study

**DOI:** 10.1186/s13014-021-01930-2

**Published:** 2021-12-04

**Authors:** Jeong Yun Jang, Su Ssan Kim, Si Yeol Song, Yeon Joo Kim, Sung-woo Kim, Eun Kyung Choi

**Affiliations:** 1 Department of Radiation Oncology, Asan Medical Center, University of Ulsan College of Medicine, 88 Olympic-ro 43-gil, Songpa-gu, Seoul, 05505 Republic of Korea; 2 Department of Radiation Oncology, Asan Medical Center, Seoul, Republic of Korea

**Keywords:** Non-small-cell lung cancer, Concurrent chemoradiotherapy, Radiation therapy, Immunotherapy, Dosimetric factor, Tumor microenvironment

## Abstract

**Background:**

Immunotherapy has been administered to many patients with non-small-cell lung cancer (NSCLC). However, only few studies have examined toxicity in patients receiving an immune checkpoint inhibitor (ICI) after concurrent chemoradiotherapy (CCRT). Therefore, we performed a retrospective study to determine factors that predict radiation pneumonitis (RP) in these patients.

**Methods:**

We evaluated the size of the planning target volume, mean lung dose (MLD), and the lung volume receiving more than a threshold radiation dose (VD) in 106 patients. The primary endpoint was RP ≥ grade 2, and toxicity was evaluated.

**Results:**

After CCRT**,** 51/106 patients were treated with ICI. The median follow-up period was 11.5 months (range, 3.0–28.2), and RP ≥ grade 2 occurred in 47 (44.3%) patients: 27 and 20 in the ICI and non-ICI groups, respectively. Among the clinical factors, only the use of ICI was associated with RP (*p* = 0.043). Four dosimetric variables (MLD, V20, V30, and V40) had prognostic significance in univariate analysis for occurrence of pneumonitis (hazard ratio, *p*-value; MLD: 2.3, 0.009; V20: 2.9, 0.007; V30: 2.3, 0.004; V40: 2.5, 0.001). Only V20 was a significant risk factor in the non-ICI group, and MLD, V30, and V40 were significant risk factors in the ICI group. The survival and local control rates were superior in the ICI group than in the non-ICI group, but no significance was observed.

**Conclusions:**

Patients receiving ICI after definitive CCRT were more likely to develop RP, which may be related to the lung volume receiving high-dose radiation. Therefore, several factors should be carefully considered for patients with NSCLC.

**Supplementary Information:**

The online version contains supplementary material available at 10.1186/s13014-021-01930-2.

## Background

Non-small-cell lung cancer (NSCLC) is one of the leading causes of cancer mortality worldwide, and approximately 25–30% of patients are initially diagnosed with stage III, which is inoperable [1, 2]. Definitive concurrent chemoradiotherapy (CCRT) was the standard treatment for locally advanced NSCLC, but it showed a short progression-free survival (PFS) of 8–11 months and overall survival (OS) of 20–23 months [3–5]. Many immunotherapy agents have been developed and have demonstrated promising results in locally advanced NSCLC patients, including immune checkpoint inhibitors (ICIs), which activate anti-tumor immune responses [6–8]. Notably, in the PACIFIC trial, the use of consolidative durvalumab after CCRT led to remarkable tumor control results. With the use of durvalumab, the median PFS was extended to 16.8 months versus 5.6 months with a placebo, and the 3-year OS rate with durvalumab and a placebo was 57.0% and 43.5%, respectively [9, 10]. Therefore, the use of adjuvant durvalumab in patients with unresectable NSCLC, whose conditions did not worsen after definitive CCRT, is now the new standard of care.

With the advent of the immunotherapy era, issues regarding the safety of treatment when administered with CCRT have emerged, although many studies have shown that the combination of ICI and CCRT is generally safe. However, in the PACIFIC trial, despite no observed significant differences, the incidence of any grade of pneumonitis was found to be 9.1% higher in the durvalumab group [9]. Radiation pneumonitis (RP) is a major complication in patients who have undergone thoracic irradiation, which can lead to a decreased quality of life, decreased performance status, and the early termination of further treatments; therefore, this needs to be carefully considered. Several studies have proven that some clinical and dosimetric factors are closely related to the occurrence of RP [11–13]. However, most have been conducted on CCRT-only groups; a few studies have targeted the patients who were additionally prescribed ICI after CCRT but only as a small cohort or as a single-arm study [14].

Therefore, we investigated the incidence of RP when ICI is used for any reason after definitive CCRT in patients with NSCLC who are unable to undergo surgery. In addition, we also sought to investigate which clinical and dosimetric factors are associated with the occurrence of RP.

## Methods

### Patients

We retrospectively reviewed the medical records of 143 consecutive NSCLC patients who underwent definitive CCRT with curative intent at Asan Medical Center from May 2018 to May 2020. Patients treated with ICI after CCRT were classified into the ICI group, and those who had never received ICI were classified into the non-ICI group. Among these patients, 37 were excluded from the analysis for the following reasons: patients with double primary cancer in the 5 years prior to the diagnosis of NSCLC (n = 7), distant metastasis at the time of diagnosis (n = 7), a history of previous lung surgery (n = 1), a history of previous thoracic radiation therapy (RT) (n = 2), radiation dose < 45 Gy (n = 1), use of ICI < 2 cycles (n = 8), and short follow-up period after CCRT of < 3 months (n = 11). Finally, 106 patients were enrolled in this study, and all of them underwent tissue biopsy, chest computed tomography (CT), 18-fluoro-deoxyglucose positron emission tomography (FDG-PET-CT), brain magnetic resonance imaging, and/or a pulmonary function test (PFT) before definitive treatment. The clinical stage of the patients was evaluated based on the 8th edition of the American Joint Committee on Cancer staging system.

### Treatments

Radiation targeted the primary tumor and clinically-involved lymph nodes. A lymph node was included in the gross tumor volume if the longest diameter was > 1 cm on its short axis by CT image and/or if the tumor had a hypermetabolic uptake on FDG-PET-CT or was proven to be a metastatic carcinoma by biopsy. No elective nodal irradiation was performed. A median total dose of 66 Gy (range, 46.0–73.0 Gy) was administered, and the fraction size was either 200 or 220 cGy. All patients received RT with the intensity-modulated radiotherapy technique. Radiation was delivered once daily (five times per week) with four to six coplanar or non-coplanar beams, and treatment verification was performed by weekly kV imaging guidance using set-up correction based on the carina and bony anatomy. The organs at risk included both lungs, the esophagus, the spinal cord, and the heart. The normal organ constraints included a maximal dose for the spinal cord of < 50 Gy, a mean lung dose (MLD) of < 20 Gy, a volume of lung receiving at least 20 Gy (V20) of < 30%, a mean esophagus dose of < 35 Gy, and a volume of heart receiving at least 60 Gy (V60) < 1/3, V45 < 2/3 and V40 < 100%. The following dosimetric parameters were generated from the dose-volume histogram (DVH) for the total lung: MLD, size of planning target volume (PTV), and the percentage of lung volume receiving more than a threshold radiation dose (VD), which refers to the relative lung volume receiving radiation above the threshold dose. As for the dose considered in VD, five variables were observed from 5 to 40 Gy (V5, V10, V20, V30, and V40).

Regarding chemotherapy, patients were prescribed paclitaxel-based paclitaxel and cisplatin (TP), paclitaxel and carboplatin (TC) regimen, an etoposide-based etoposide and cisplatin (EP), etoposide and carboplatin regimen, or cisplatin with pemetrexed. Of 106 patients, 51 received ICI after CCRT (Additional File 1). As the analysis was performed regardless of the treatment aim, all patients with consolidative, salvage, and palliative aims were enrolled together. PD-1/PDL-1 inhibitors were used as ICIs, including durvalumab, atezolizumab, pembrolizumab, and nivolumab. Durvalumab was prescribed at 10 mg /kg every 2 weeks, atezolizumab at 1200 mg every 3 weeks, pembrolizumab at 200 mg every 3 weeks, and nivolumab at 3 mg/kg every 2 weeks (all injected intravenously). All ICIs were discontinued when patients were determined to be intolerant due to adverse events or progressive disease.

### Follow-up and outcomes

During the CCRT period, chest X-rays (CXR) and complete blood counts were performed weekly. All patients were regularly followed up by a radiation oncologist and a medical oncologist after the termination of CCRT. At each follow-up, history taking, physical examination, CXR, and blood tests were performed. Chest CT was performed every 6 months for 2 years and annually for 5 years.

The primary endpoint was RP ≥ grade 2. RP was assessed according to the toxicity criteria of the lung fibrosis established by Radiation Therapy Oncology Group and the European Organization for Research and Treatment of Cancer. The definitions were as follows: Grade 1, with no symptoms such as cough or shortness of breath and/or no opacity changes on chest CT; Grade 2, with moderate symptoms which require outpatient steroid treatment and/or patchy image changes; Grade 3, with severe symptoms which need hospitalization and/or increased density imaging changes; Grade 4, with severe symptoms requiring continuous O_2_ or assisted ventilation; Grade 5, expired. Based on the readings of the experienced chest radiologist, the RP was assessed by two radiation oncologists blindly on whether ICI was administered or not. Otherwise, all toxicity excluding RP, was assessed according to the National Cancer Institute’s Common Terminology Criteria for Adverse Events ver. 5.0. The secondary endpoints were survival rate, locoregional failure (LRF) rate, distant metastasis (DM) rate, and acute and late toxicity. The LRF rate was evaluated by dividing cases into in-field and out-of-field failures based on whether the failure occurred inside or outside the PTV field. Recurrence in the ipsilateral lung was considered as LRF, and nodules in the contralateral lung or any pleural metastasis were considered as DM.

### Statistical analysis

Statistical analyses were performed using SPSS version 22.0 (SPSS Inc., Chicago, IL, USA) and R version 3.6.1 (web-r.org). To compare the distribution of patient, disease, and treatment characteristics between the ICI group and non-ICI group, an independent-sample t-test was performed for continuous variables and chi-square test was performed for non-continuous variables. A receiver operating characteristic (ROC) curve was drawn to evaluate how well each DVH parameter could distinguish the incidence of RP, and through this, a suitable cut-off value and significance probability were calculated. Based on the cut-off values estimated from here, univariate and multivariate analyses for the occurrence of pneumonitis for the ICI and non-ICI groups were assessed with Fine and Gray competing risk regression analysis. P-values below 0.05 were considered statistically significant.

## Results

### Patients’ characteristics

One hundred and six patients were included, and their characteristics are listed in Table 1. The median age of the patients was 64 years (range, 38–82), and 84% were male. Most patients showed a good Eastern Cooperative Oncology Group Performance Status of 0–1. The mean values of PFT did not differ significantly between the two groups. Approximately half of the patients with squamous cell carcinoma and adenocarcinoma were enrolled in the study. Most of the recruited patients (90.6%) were stage III. The characteristics that showed differences between the two groups were age at the time of diagnosis, diffusing capacity of the lung for CO, tumor location, and the type of chemotherapy agent used during CCRT, but none of these factors was a significant factor in the development of pneumonitis, as shown in subsequent analysis.

**Table 1:** Patient and disease characteristics

Characteristics	Total (n = 106)		ICI (n = 51)		Non-ICI (n = 55)		*p*^†^
Median age (median, [range])	64 (38–82)		62 (36–76)		66 (38–82)		0.004
Sex (n [%])							0.051
Male	89 (84.0)		40 (78.4)		49 (89.1)		
Female	17 (16.0)		11 (21.6)		6 (10.9)		
ECOG PS before CCRT (n [%])							0.570
0–1	97 (91.5)		45 (88.2)		52 (94.5)		
2	7 (6.6)		5 (9.8)		2 (3.6)		
3	2 (1.9)		1 (2.0)		1 (1.8)		
ECOG PS before ICI (n [%])							
0–1			45 (88.2)				
2			3 (5.9)				
Unavailable			3 (5.9)				
Underlying lung disease (n [%])	21 (19.8)		9 (17.6)		12 (21.8)		0.087
ILD	1 (0.9)		1 (2.0)		0 (0.0)		
COPD	13 (12.3)		4 (7.8)		9 (16.4)		
Asthma	4 (3.8)		0 (0.0)		4 (7.3)		
TB	8 (7.5)		4 (7.8)		4 (7.3)		
Smoking status (n [%])							0.255
(Ex-) smoker	82 (77.4)		37 (72.5)		45 (81.8)		
Never-smoker	24 (22.6)		14 (27.5)		10 (18.2)		
PFT (mean, [range])							
FVC, %	83.0 (54.0–118.0)		85.0 (57.0–118.0)		82.0 (54.0–110.0)		0.202
FEV1, L	2.00 (0.89–3.61)		3.00 (0.89–3.57)		2.00 (1.05–3.61)		0.076
FEV1, %	76.0 (24.0–118.0)		78.0 (24.0–118.0)		74.0 (33.0–114.0)		0.221
DLCO, %	75.0 (36.0–124.0)		81.0 (48.0–132.0)		71.0 (36.0–124.0)		0.013
Tumor histology (n [%])							0.710
SqCC	52 (49.1)		22 (43.1)		30 (54.5)		
Adenocarcinoma	50 (47.2)		29 (56.9)		21 (38.2)		
Others	4 (3.8)		0 (0.0)		4 (7.3)		
Tumor location (n [%])							
Right upper lobe	43 (40.6)		26 (51.0)		17 (30.9)		0.018
Right middle lobe	4 (3.8)		3 (5.9)		1 (1.8)		
Right lower lobe	15 (14.2)		5 (9.8)		10 (18.2)		
Left upper lobe	32 (30.2)		14 (27.5)		18 (32.7)		
Left lower lobe	12 (11.3)		3 (5.9)		9 (16.4)		
Stage (n [%])							0.657
IIA	10 (9.4)		5 (9.8)		5 (9.1)		
IIIA	27 (25.5)		10 (19.6)		17 (30.9)		
IIIB	53 (50.0)		29 (56.9)		24 (43.6)		
IIIC	16 (15.1)		7 (13.7)		9 (16.4)		
EGFR mutation (n [%])							
Positive	11 (10.4)		5 (9.8)		6 (10.9)		0.334
Negative	36 (34.0)		20 (39.2)		16 (29.1)		
Not checkable	59 (55.7)		26 (51.0)		33 (60.0)		
PD-L1 test (n [%])							
Positive			36 (70.6)				
Negative			8 (15.7)				
Not checkable			7 (13.7)				
Chemotherapy agent during CCRT (n [%])							
Paclitaxel/ Cisplatin	71 (67.0)		42 (82.4)		29 (52.7)		0.022
Paclitaxel/ Carboplatin	15 (14.2)		3 (5.9)		12 (21.8)		
Etoposide/ Cisplatin	14 (13.2)		4 (7.8)		10 (18.2)		
Etoposide/ Carboplatin	5 (4.7)		2 (3.9)		3 (5.5)		
Cisplatin/ Pemetrexed	1 (0.9)		0 (0.0)		1 (1.8)		
No. of cycles of chemotherapy (median, [range])	6 (2–8)		6 (2–7)		6 (2–8)		0.233
Dosimetric parameters							
Total dose (median, [range])	66.0 (46.0–73.0)		66.0 (46.0–69.0)		66.0 (54.0–73.0)		0.441
PTV size, cm^3^ (median, [range])	317.6 (104.8–1250.6)		329.9 (136.8–1250.6)		296.6 (104.8–847.2)		0.216
MLD, Gy (median, [range])	13.1 (2.7–21.6)		13.3 (2.7–21.3)		12.1 (3.9–21.6)		0.919
V5, %, (median, [range])	45.8 (7.5–83.5)		46.8 (7.5–83.4)		45.3 (12.7–83.5)		0.557
V10, %, (median, [range])	33.2 (5.8–61.4)		33.2 (5.8–59.9)		32.8 (9.5–61.4)		0.611
V20, %, (median, [range])	22.3 (3.2–45.5)		22.6 (3.2–45.5)		20.9 (7.1–41.4)		0.982
V30, %, (median, [range])	16.3 (1.1–36.5)		17.5 (1.1–29.3)		15.8 (3.6–36.5)		0.705
V40, % (median, [range])	12.3 (0.3–25.1)		13.2 (0.3–25.1)		11.2 (2.0–24.0)		0.399

Values are number (percentage) or median (range). Because of rounding, not all percentages total 100

*ECOG PS* Eastern Cooperative Oncology Group performance status, *CCRT* Concurrent chemoradiotherapy, *ICI* Immune checkpoint inhibitor, *ILD* Interstitial lung disease, *COPD* Chronic obstructive pulmonary disease, *TB* Tuberculosis, *PFT* Pulmonary function test, *FVC* Forced vital capacity, *FEV1* Forced expiratory volume in 1 s, *DLCO* Diffusing capacity of the lung for CO, *SqCC* Squamous cell carcinoma, *EGRF* Epidermal growth factor receptor, *PD-L1* Programmed death-ligand 1, *PTV* Planning target volume, *MLD* Mean lung dose, *VD (V5, V10, V20, V30, V40)* The percentage of lung volume receiving more than a threshold radiation dose (5, 10, 20, 30, 40 Gy)

^†^Significance value for the difference in the distribution of the ICI and non-ICI groups

### Univariate and multivariate analyses for radiation pneumonitis

**Fig. 1:**
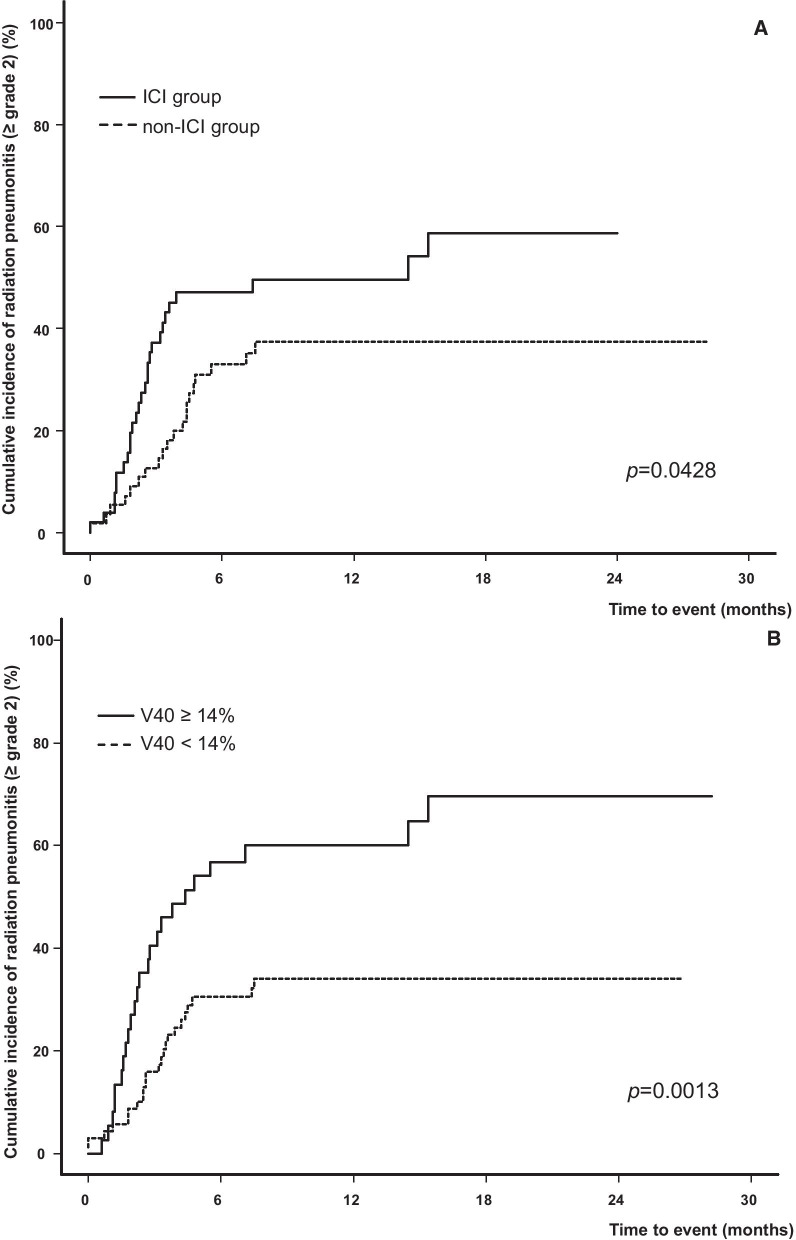
Cumulative incidence of radiation pneumonitis ≥ grade 2 according to time to event between **a** ICI and non-ICI groups and **b** V40 ≥ 14% and < 14%. *ICI* Immune checkpoint inhibitor. *V40* The percentage of lung volume receiving more than 40 Gy

The median follow-up period was 11.5 months (range, 3.0–28.2). During this period, RP ≥ grade 2 occurred in 47 patients (44.3%), and six (5.7%) presented with RP ≥ grade 3. Univariate analysis was performed to determine the prognostic factors predicting RP ≥ grade 2. The results are shown in Table 2, and among the clinical factors, only the use of ICI was confirmed as a significant factor (*p* = 0.043). For the dosimetric factor, a cut-off value was set, and the hazard ratio (HR) was derived for the occurrence of pneumonitis, by dividing the group into patients with a value higher or lower than this. In univariate analysis for the entire cohort based on the cut-off value derived here, at an MLD of 16 Gy, V20, V30, and V40 showed a significant HR, which might increase the occurrence of RP (MLD, *p* = 0.009; V20, *p* = 0.007; V30, *p* = 0.004; V40, *p* = 0.001). Figure 1 shows the cumulative incidence according to the use of ICI and the size of V40, which was the most significant factor among various dosimetric factors. In multivariate analysis, the use of ICI, MLD, and V40 were identified as significant factors. However, values from V5 to V40 and MLD were highly correlated with each other, so they were not included in one analysis model as covariates and examined separately. Also, only V40, which had the highest significance, was selected for analysis on behalf of several VD values (Additional File 2).

**Table 2:** Clinical and dosimetric factors predicting radiation pneumonitis ≥ grade 2 for patients treated with concurrent chemoradiotherapy

Variable	HR (95% CI)		*p*	Comparison group
**Clinical factors**				
Age, ≥ 65 years	1.136 (0.644–2.004)		0.660	< 65
ECOG PS before CCRT, ≥ 2	0.421 (0.102–1.744)		0.233	< 2
ECOG PS before ICI, ≥ 2^†^	1.754 (0.360–8.541)		0.487	
(Ex-) smoker	1.356 (0.698–2.633)		0.369	Never-smoker
Underlying lung disease	1.162 (0.587–2.300)		0.668	No underlying lung disease
EGFR mutation	1.155 (0.445–2.998)		0.767	Wild type
PFT				
FVC, %	0.992 (0.973–1.1012)		0.428	[Continuous]
FEV1, L	0.930 (0.579–1.493)		0.763	[Continuous]
FEV1, %	0.991 (0.977–1.006)		0.255	[Continuous]
DLCO, %	0.995 (0.980–1.010)		0.487	[Continuous]
Clinical stage, ≥ IIIA	1.358 (0.503–3.664)		0.546	Stage II
Tumor location, lower lobe	2.014 (0.951–4.266)		0.067	Tumor at RUL, RML, LUL
Use of ICI	1.802 (1.019–3.186)		0.043	Non-ICI group
Chemotherapy agent during CCRT, Paclitaxel/ Cisplatin	1.498 (0.834–2.690)		0.177	Chemotherapy agent other than Paclitaxel/ Cisplatin
No. of cycle of chemotherapy	0.903 (0.764–1.068)		0.234	[Continuous]
Dosimetric factors				
Total dose, > 60 Gy	1.084 (0.615–1.910)		0.782	≤ 60 Gy
PTV volume, ≥ 280 cm^3^	1.459 (0.815–2.612)		0.204	< 280 cm^3^
MLD, ≥ 16 Gy	2.299 (1.234–4.284)		0.009	< 16 Gy
V5, ≥ 28%	2.359 (0.880–6.322)		0.088	< 28%
V10, ≥ 23%	1.914 (0.851–4.304)		0.116	< 23%
V20, ≥ 19%	2.898 (1.331–6.309)		0.007	< 19%
V30, ≥ 18%	2.295 (1.297–4.060)		0.004	< 18%
V40, ≥ 14%	2.513 (1.431–4.412)		0.001	< 14%

*HR* Hazard ratio, *CI* confidence interval, *ECOG PS* Eastern Cooperative Oncology Group performance status, *CCRT* Concurrent chemoradiotherapy, *ICI* Immune checkpoint inhibitor, *EGRF* Epidermal growth factor receptor, *PFT* Pulmonary function test, *FVC* Forced vital capacity, *FEV1* Forced expiratory volume in 1 s, *DLCO* Diffusing capacity of the Lung for CO, *RUL* Right upper lobe, *RML* Right middle lobe, *LUL* Left upper lobe, *PTV* Planning target volume, *MLD* Mean lung dose, *VD (V5, V10, V20, V30, V40)* The percentage of lung volume receiving more than a threshold radiation dose (5, 10, 20, 30, 40 Gy)

^†^Only 51 patients from the ICI group

### Radiation pneumonitis in ICI and non-ICI groups

The incidence of RP ≥ grade 2 was 52.9% for the ICI group, which was approximately 16% higher than that of the non-ICI group (*p* = 0.043) (Table 3). Severe RP ≥ grade 3 occurred in three patients in both groups, and the incidence in the ICI and non-ICI groups was 5.9% and 5.4%, respectively (*p* = 0.924). The interval between the end of CCRT and the occurrence of complications was 2.3 months for the ICI group and 3.7 months for the non-ICI group, with no significant difference (*p* = 0.782). In addition, the duration of steroid use due to pneumonitis was 56 days in the ICI group and 67 days in the non-ICI group, which was also similar in both groups with no significant difference (*p* = 0.848).

**Table 3:** Characteristics of radiation pneumonitis according to immune checkpoint inhibitor administration

Characteristics	ICI (n = 51)	Non-ICI (n = 55)	*p*
Patients with RP ≥ grade 2 (n [%])	27 (52.9)	20 (36.4)	0.043
Patients with RP ≥ grade 3 (n [%])	3 (5.9)	3 (5.4)	0.924
Onset time from end of CCRT, month (median, [range])^†^	2.3 (0.0–15.4)	3.7 (0.0–7.5)	0.782
Onset time from the start of ICI, month (median, [range])^†^	1.4 (0.0–5.8)	–	–
Duration of steroid use, day (median, [range])^†^	56 (16–238)	67 (6–114)	0.848

*RP* Radiation pneumonitis, *ICI* Immune checkpoint inhibitor, *CCRT* Concurrent chemoradiotherapy

^†^Only for patients with RT pneumonitis ≥ grade 2

**Fig. 2:**
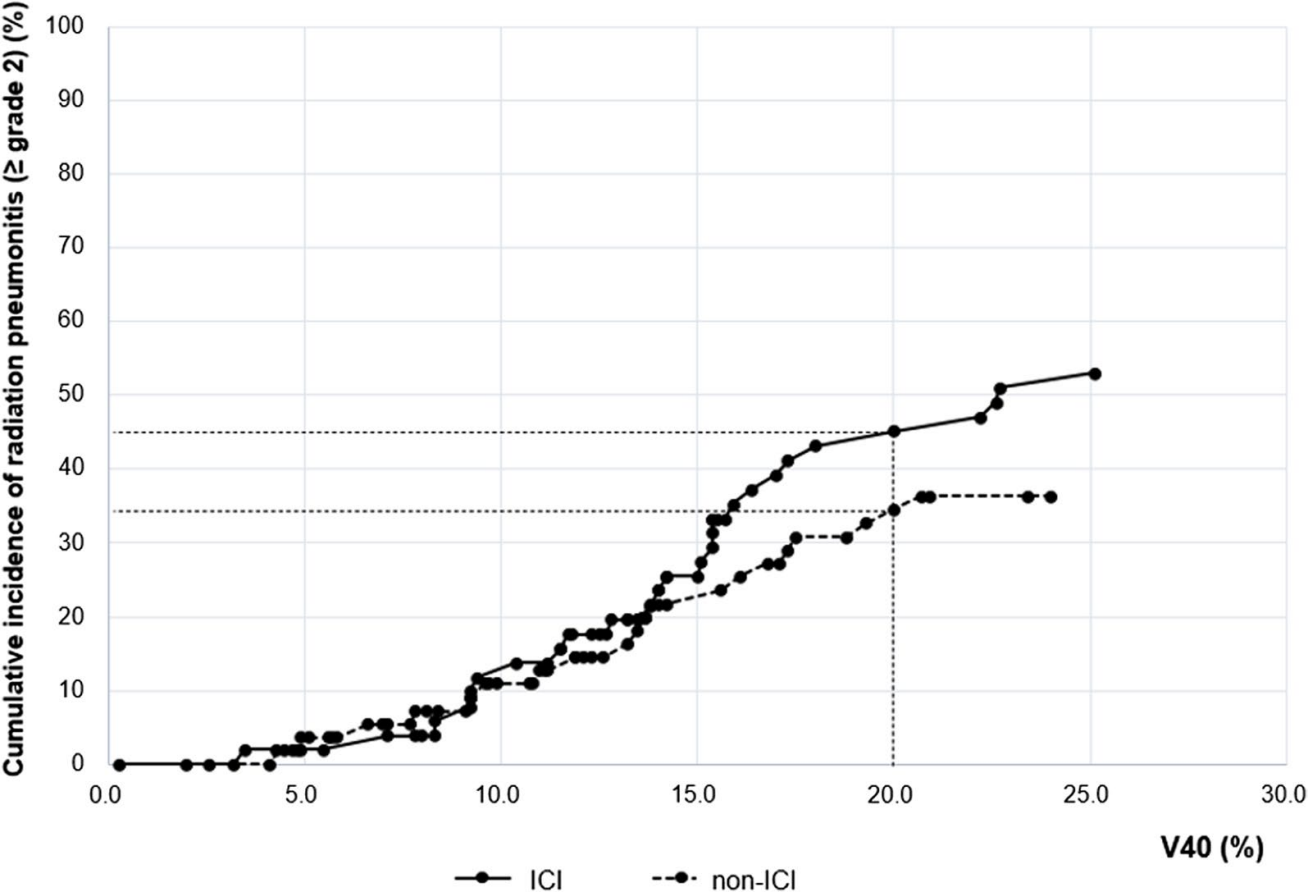
Cumulative incidence of radiation pneumonitis ≥ grade 2 by V40. *ICI* Immune checkpoint inhibitor

To establish the dosimetric factors that can predict the RP in each group, a univariate analysis was performed with the same cut-off value used for the entire cohort, and the results are shown in Table 4. In the ICI group, at an MLD of 16 Gy, V30 and V40 could significantly predict the occurrence of RP, and in the non-ICI group, only V20 could significantly predict RP. Therefore, we plotted the cumulative incidence of RP with V40, which showed the most significant relationship in the ROC curve and univariate analysis. It was found that the higher the value of V40, the greater the difference in the cumulative incidence between the two groups (Fig. 2). The cumulative incidence of patients with a V40 value of 0–20% was 45.1% in the ICI group and 34.5% in the non-ICI group, showing a difference of approximately 10.6%.

**Table 4:** Dosimetric factors predicting radiation pneumonitis ≥ grade 2 for patients treated with concurrent chemoradiotherapy using ICI

Variable	ICI (n = 51)			Non-ICI (n = 55)			*p* for interaction^†^
	HR (95% CI)		*p*	HR (95% CI)		*p*	
Total dose, > 60 Gy	1.098 (0.507–2.377)		0.813	1.124 (0.480–2.634)		0.787	0.967
PTV volume, ≥ 280 cm^3^	1.001 (0.456–2.198)		0.997	1.968 (0.819–4.729)		0.130	0.260
MLD, ≥ 16 Gy	5.030 (1.886–13.416)		0.001	1.485 (0.601–3.671)		0.392	0.074
V5, ≥ 28%	3.212 (0.773–13.345)		0.108	1.741 (0.438–6.925)		0.431	0.546
V10, ≥ 23%	1.921 (0.652–5.662)		0.237	1.907 (0.561–6.488)		0.301	0.993
V20, ≥ 19%	2.177 (0.739–6.411)		0.158	3.231 (1.071–9.748)		0.037	0.615
V30, ≥ 18%	2.533 (1.155–5.557)		0.020	2.054 (0.882–4.783)		0.095	0.721
V40, ≥ 14%	2.754 (1.249–6.070)		0.001	1.964 (0.851–4.533)		0.114	0.566

*ICI* Immune checkpoint inhibitor, *HR* Hazard ratio, *CI* Confidence interval, *PTV* Planning target volume, *MLD* Mean lung dose, *VD (V5, V10, V20, V30, V40)* The percentage of lung volume receiving more than a threshold radiation dose (5, 10, 20, 30, 40 Gy)

^†^Interaction effect; the effect of each variable is significantly different between ICI and non-ICI group

We also examined whether the interval between the end of CCRT and the start of immunotherapy in the ICI group affected the occurrence of RP. As a result of comparing the incidence of pneumonitis with an interval of 3 months, 51.4% of those who started ICI within 3 months of CCRT and 57.1% of those who started after 3 months had RP ≥ grade 2, showing no significant difference (*p* = 0.712).

### Survival and toxicities other than radiation pneumonitis

The survival and patterns of recurrence are shown in Additional File 3. In this analysis, only 90 patients were included; we excluded 16 patients who received immunotherapy with palliative and salvage aim within the ICI group. Of the 90 patients, 15 died, with a crude survival rate of 83.3%. LRF occurred in 27 patients (30.0%), and DM occurred in 17 patients (18.9%): 10 in the ICI group and 7 in the non-ICI group. The most frequent metastasis sites were the bone, the liver, and the brain, in order of frequency.

Toxicity data for all patients are shown in Additional File 4. For acute toxicity, 58 events occurred in 40 patients within the ICI group, and 39 events occurred in 23 patients within the non-ICI group. RP occurred most frequently, followed by esophagitis and dermatitis. In terms of late toxicity, 19 events occurred in 19 patients in the ICI group, and 21 events occurred in 21 patients in the non-ICI group. RP occurred most frequently, and peripheral neuropathy and pneumothorax were rare complications. Ten out of 106 patients (9.4%) had acute toxicity ≥ 3 grade, and three patients (2.8%) had late toxicity ≥ grade 3. Most patients with acute toxicity ≥ grade 3 recovered after various treatments such as antibiotics, steroid treatment, or esophageal stent insertion, but one patient who suffered brachial plexopathy did not improve despite the rehabilitation program. All three patients with late toxicity ≥ grade 3 experienced RP and seemed to improve with inpatient treatment, but two of them died from combined pneumonia and poor lung function.

## Discussion

Several small cohort studies have reported the occurrence of RP in patients with NSCLC after the use of ICI with CCRT. In a study by Narek et al., RP ≥ grade 2 occurred in 18% of patients using durvalumab after CCRT, which was significantly higher than that of the CCRT-only group, which was only 9% (*p* = 0.09) [15]. A single-arm study by Moore et al. found that 54% of patients who took durvalumab after CCRT had RP ≥ grade 2 [14]. In our study, the incidence was 16.5% higher in the ICI group than the non-ICI group, showing a similar tendency to the previous studies (ICI group 52.9% vs. non-ICI group 36.4%, *p* = 0.043). In the PACIFIC trial, any grade of RP was 9.1% higher in the ICI group (durvalumab group 33.9% vs. placebo group 24.8%), and the difference in the incidence of RP between the treatment groups was slightly greater in our study. However, for RP ≥ grade 3, similar incidence rates were observed between the two groups in both our study and the PACIFIC study [16].

In general, RP ≥ grade 2 is known to occur in 10–40% of patients with NSCLC who undergo CCRT with conventional fraction [17]. In our study, 44.3% of patients developed RP ≥ grade 2, a higher incidence than that observed in other studies. There are likely to be several reasons for this result. First, the PACIFIC trial subgroup analysis found that Asians and patients with EGFR mutations had a high incidence of RP [16]. As the participants in our study were all Koreans, racial factors could explain the high RP incidence. Second, a high percentage of patients had EGFR mutations in this study; even if the mutation itself was not identified as a risk factor in univariate analysis in this study, in the PACIFIC trial, mutations were found in 8.2% of those tested and in 23.4% in this study. Third, patients who received neoadjuvant chemotherapy before CCRT were not included in this study. If the tumor size is reduced through induction chemotherapy, it can be expected that the radiation administered to the normal lung can also be reduced. In the PACIFIC trial, approximately a quarter of patients underwent induction treatment before CCRT. Finally, the high incidence of RP in the current study may be due to the early initiation of steroid therapy when the patient complained of a mild cough or dyspnea, which might be less associated with RP.

Some studies have indicated that clinical factors, including the presence of chronic lung disease, stage IIIA or higher disease, tumors located in the lower lobe, and previous history of RT, can significantly increase the probability of RP ≥ grade 2 [13]. However, none of these were found to be associated with the occurrence of RP in our study. Among clinical factors, only the use of ICI was found to be a significant factor. On the other hand, we found that initiating ICI at any point after the end of CCRT did not affect the development of RP; thus, it would be unnecessary to deliberately initiate immunotherapy late due to concerns about the potential side effects.

In our study, only V20 was identified as a factor correlated with RP in the non-ICI group. However, in the ICI group, significant HR were shown with V30 and V40, and the incidence of RP was found to increase in the high VD area. Usually, the lung volume irradiated with a high dose tends to correlate with target size. However, when observing the correlation between the PTV size and RP, the HR was 1.459, and there was no significant difference between the two groups; thus, V30 and V40 may have had a relationship with RP distinct from the field size. In addition, a similar study by Satoshi et al. also found that V30 had the largest AUC among various parameters in patients receiving adjuvant durvalumab after CCRT [18]. Previous studies have shown that sufficient lymphocytic infiltration is achieved through RT, which turns “cold” tumors “hot” and creates a tumor microenvironment (TME) that can respond well to various ICIs [19]. However, there remains controversy regarding the optimal dose and fraction scheme that can induce a change in the TME, and no definite conclusions have been drawn. In a preclinical study based on NSCLC cell line, the most potent T cell infiltration was induced by high-dose radiation; according to another study by Marconi et al., the probability of the occurrence of the abscopal effect based on changes in the immunologic environment is 50% with a biologically effective dose of at least 60 Gy [20, 21]. Therefore, there is a high possibility that a “hot” tumor environment will be created in the high dose area, which may be beneficial in terms of tumor cell control but is also the reason for the increased incidence of RP.

The survival and local control rate were superior in the ICI group, showing similar trends as those reported in the PACIFIC trial. However, these results were not significant, and we predict that this is due to an insufficient follow-up period of < 1 year. In addition, patients with varying responses after CCRT were recruited into one group despite receiving different types of ICI, which makes it difficult to compare oncological outcomes directly. Future studies should be conducted on a larger number of patients with more uniform conditions and treatment characteristics, after which it is expected that results similar to those of other prospective studies will be obtained.

The limitations of this study were the small number of patients and the short follow-up time. Additionally, owing to the study's retrospective nature, the patient characteristics were somewhat different. Nonetheless, it would be of great significance to establish the frequency of adverse events in the new treatment combination and which factors affect them in the actual clinical situation.

## Conclusions

The use of ICI after definitive CCRT is generally safe. However, it is important to be aware that the incidence of RP ≥ grade 2 is slightly higher after ICI, and as this is associated with the lung volume irradiated with high doses of radiation, this should be fully considered in the treatment planning process for NSCLC patients.

## Supplementary Information

**Additional file 1**. Treatment characteristics of the ICI group.

**Additional file 2**. Multivariate analysis results using fine and gray competing risk regression analysis.

**Additional file 3**. Oncologic outcomes for 90 patients with non-small cell lung cancer treated with concurrent chemoradiotherapy.

**Additional file 4**. Toxicity results for 106 patients with non-small cell lung cancer treated with concurrent chemoradiotherapy.

## Data Availability

The data that support the findings of this study are available on request from the corresponding author.

## Abbreviations

NSCLC: Non-small-cell lung cancer
CCRT: Concurrent chemoradiotherapy
PFS: Progression-free survival
OS: Overall survival
ICIs: Immune checkpoint inhibitors
RP: Radiation pneumonitis
RT: Radiation therapy
CT: Computed tomography
FDG-PET-CT: 18-Fluoro-deoxyglucose positron emission tomography
PFT: Pulmonary function test
MLD: Mean lung dose
VD: Percentage of lung volume receiving more than a threshold dose
DVH: Dose-volume histogram
PTV: Planning target volume
CXR: Chest X-ray
LRF: Locoregional failure
DM: Distant metastasis
ROC: Receiver operating characteristic
HR: Hazard ratio
TME: Tumor microenvironment
